# Human nasal epithelial cells express IL-5Rα but not the co-receptor CSF2RB and do not signal to IL-5

**DOI:** 10.1111/all.16218

**Published:** 2024-07-07

**Authors:** Aaqib Sohail, Laura Cho, Jonathan Hacker, Regan W. Bergmark, Stella E. Lee, Alice Maxfield, Rachel E. Roditi, Daniel F. Dwyer, Kathleen M. Buchheit, Tanya M. Laidlaw

**Affiliations:** 1 Department of Medicine, Harvard Medical School, Boston, Massachusetts, USA; 2 Division of Allergy and Clinical Immunology, Brigham and Women’s Hospital, Boston, Massachusetts, USA; 3 Department of Surgery, Division of Otolaryngology-Head & Neck Surgery, Brigham and Women’s Hospital, Harvard Medical School, Boston, Massachusetts, USA

## To the Editor,

IL-5 plays a crucial role in type 2 inflammation, thought to be primarily associated with eosinophil-related inflammation; however, it is not clear whether IL-5 plays any relevant role in the initiation of or contribution to epithelial cell-driven allergic responses. IL-5 exerts biological effects by binding to its receptor, IL-5 receptor alpha (IL-5Rα), which forms a complex with the common beta-chain receptor, CSF2RB, to initiate intracellular signaling.^1^ The IL-5Rα is also expressed in non-eosinophil cells of the respiratory tract, including some antibody-secreting cells, mast cells, and epithelial cells (EpC),^2^ but the impact of IL-5 on non-eosinophil cells, especially in the upper respiratory tract, remains poorly understood. Further, although anti-IL-5 treatment does decrease blood and sputum eosinophils, it does not decrease the late allergen-induced asthmatic response or the airway hyper-responsiveness to histamine, which calls into question the role of eosinophils, and of IL-5 itself, in mediating asthma and causing airway hyper-responsiveness.^3^ In 2020, Barretto et al. reported that differentiated human bronchial EpCs express both functional IL-5Rα and CSF2RB,^4^ suggesting an additional cell type that may be relevant in asthma and affected by IL-5 stimulation or anti-IL-5 treatments; however, the relevance of IL-5 to the EpCs of the upper respiratory tract was not known. Interestingly, trials with IL-5/5Rα-targeted therapies have demonstrated efficacy in eosinophilic asthma but have yielded mixed results in terms of efficacy in the upper respiratory tract in chronic rhinosinusitis with nasal polyps (CRSwNP).^5^ In this study, we aimed to provide a comprehensive analysis of the expression profiles and potential functions of IL-5 receptors in sinonasal EpCs. We present novel data demonstrating that while differentiated sinonasal EpCs express IL-5Rα, they lack the CSF2RB co-receptor required for IL-5 signaling. This dichotomy between upper airway sinonasal and lower airway bronchial EpCs suggests tissue-specific differences in IL-5 responsiveness, with implications for understanding respiratory diseases and the development of targeted therapies.

**FIGURE 1:**
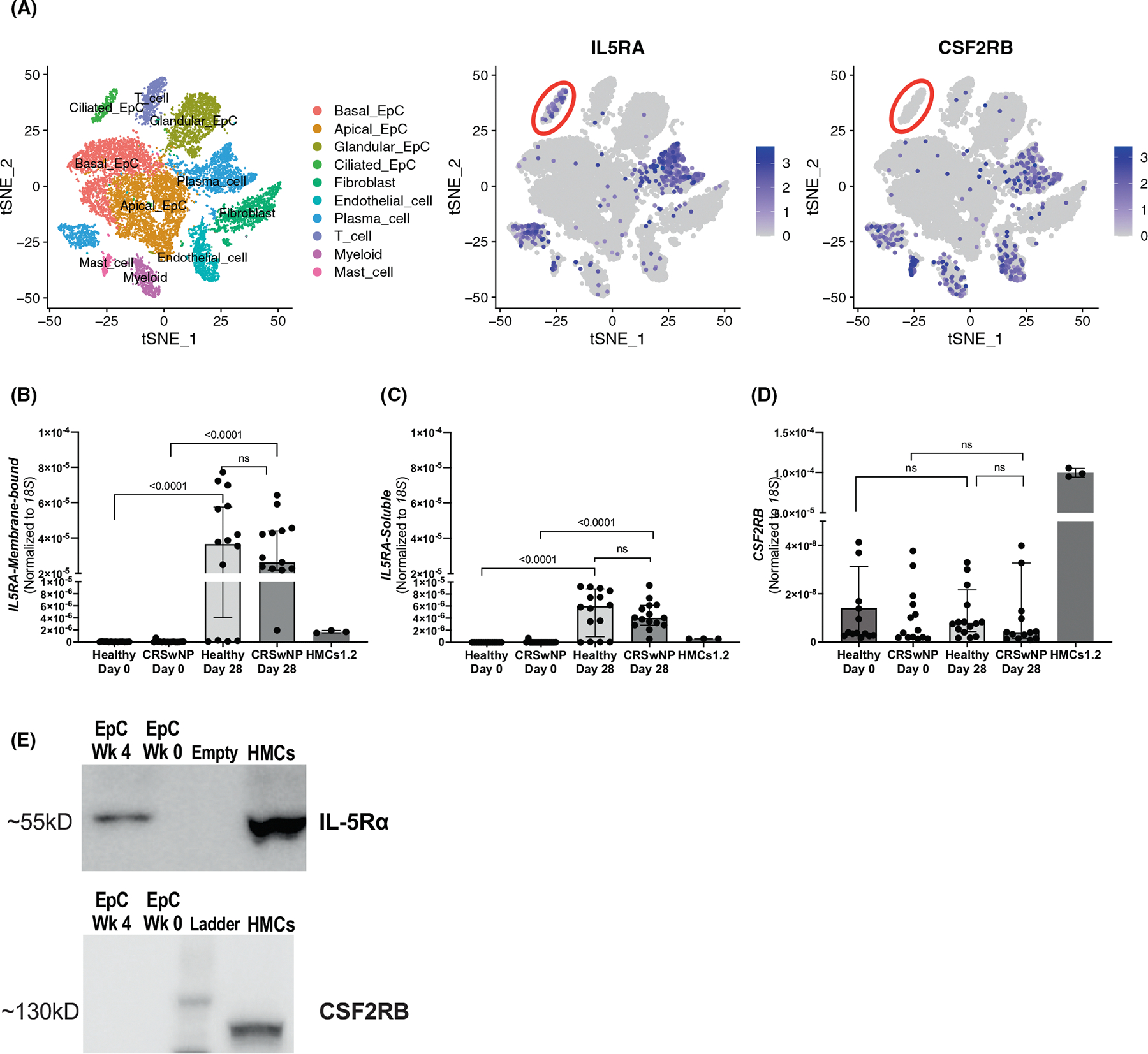
Expression of the two subunits of the IL-5 receptor in human sinonasal epithelial cells. Single-cell RNA-seq data illustrating the expression of *IL5RA* (left) and *CSF2RB* (right) in surgically excised nasal polyp tissue cells (A). Quantitative PCR of the expression of membrane-bound (B) and soluble (C) variants of *IL5RA*, and *CSF2RB* (D) in sinonasal epithelial cells (EpCs) from healthy controls (*n* = 14) or patients with chronic rhinosinusitis with nasal polyps (CRSwNP) (*n* = 15) cultured over 4 weeks of differentiation and the positive control HMC-1.2 human mast cell line. Individual points are shown, along with median with interquartile range. Representative western blots showing the expression of IL-5Rα and CSF2RB in cultured sinonasal EpCs and the HMC-1.2 (E).

Demographic characteristics for the study participants from whom samples were collected is shown in Table S1. Single-cell RNA sequencing data from respiratory tissue of eosinophilic (“type 2 high”) CRSwNP and CRS non-polyp patients revealed that among EpCs, *IL5RA* is expressed nearly exclusively in the ciliated epithelium (Figure 1A). However, the beta subunit of the IL-5 receptor, *CSF2RB* was not robustly expressed in any sinonasal EpCs (Figure 1A). To explore this, we established an in vitro sinonasal tissue EpC differentiation model using air-liquid interface (ALI) to culture EpCs from CRSwNP patients and healthy donors.

To determine the levels of two common *IL5RA* variants^6^ and *CSF2RB* in the submerged (Day 0) and ALI-differentiated (Day 28) sinonasal EpC cultures, we used variant-specific qPCR primers. Results revealed that the dominantly expressed transcript was the membrane-bound IL5RA-M form, suggesting the capacity to signal (Figure 1B,C). However, *CSF2RB* transcript was essentially undetectable by qPCR in sinonasal EpCs from both patient groups (Figure 1D; Figure S1). Western blot analysis for the two receptors confirmed the qPCR results at protein levels (Figure 1E).

**FIGURE 2:**
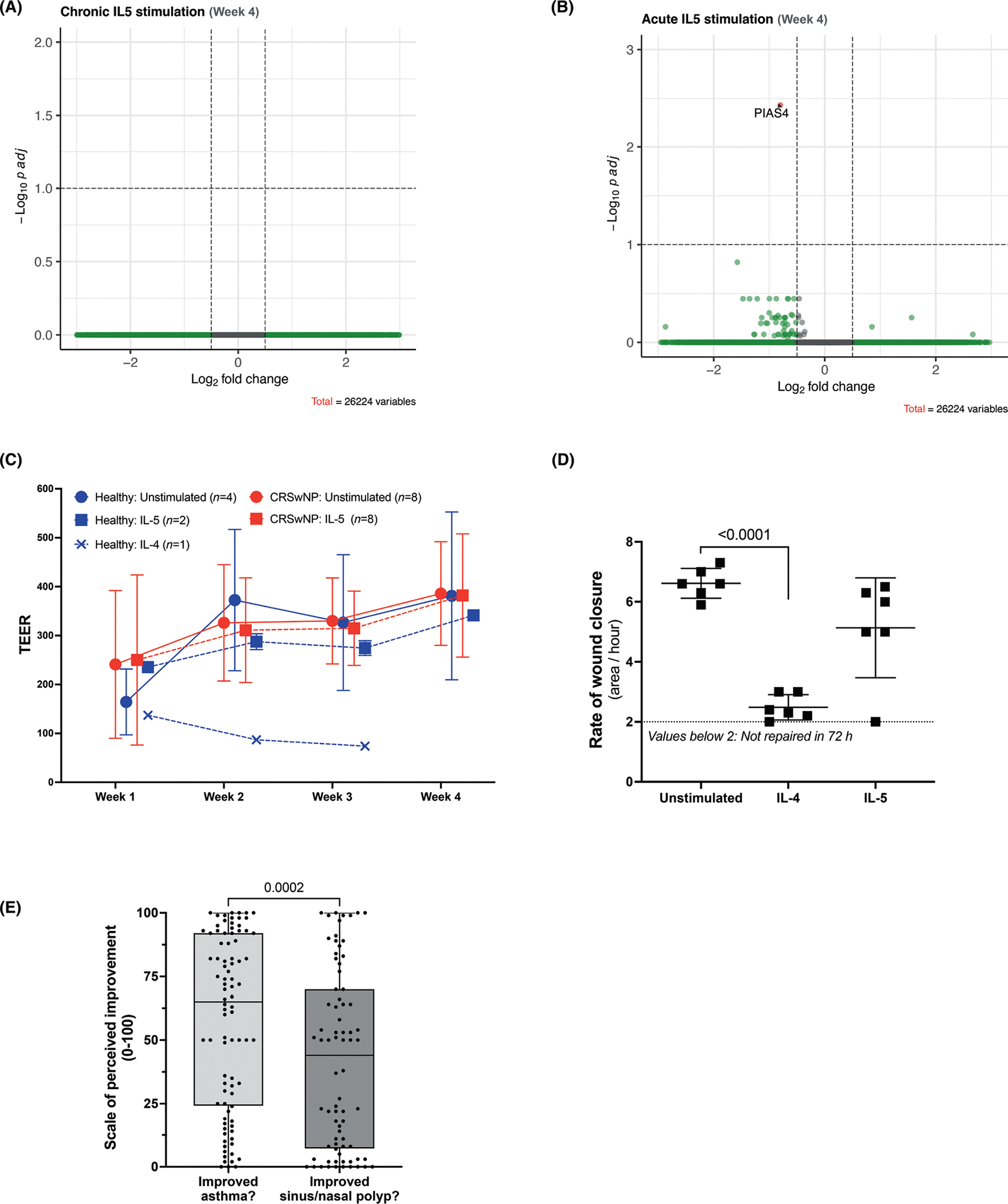
Effect of IL-5 on differential transcript expression of cultured human sinonasal tissue epithelial cells. Differential expression analysis between chronically IL-5 (10 ng/mL) stimulated (4 weeks) and unstimulated cells (A) and between acutely IL-5 (10 ng/mL) stimulated (×24 h) and unstimulated cells (B). Transepithelial electrical resistance (TEER) of cultured sinonasal tissue epithelial cells was measured at 4 timepoints in response to stimulation with IL-5 (10 ng/mL) or IL-4 (10 ng/mL) (C). The rate of wound closure was calculated by determining the ratio of the repaired wound area to the time taken for repair (*n* = 6) (D). Patient-reported efficacy of anti-IL-5 monoclonal antibody for treatment of lower respiratory (asthma) and upper respiratory (nasal polyps) symptoms. Survey data results from 89 patients with asthma and CRSwNP who had been treated with the anti-IL-5 biologic mepolizumab for at least 3 months. All data points are shown, with boxes showing median and interquartile range (E).

Despite the lack of co-receptor expression, we looked for possible IL-5-induced signaling functions on the sinonasal EpCs. We either stimulated 28-day differentiated EpCs with IL-5 for 24 h or added IL-5 into the culture media for the entire 4 weeks of culture to study acute and chronic effects, respectively, on changes in transcript expression, transepithelial electrical resistance (TEER), and self-tissue repair. Bulk RNAseq of those samples revealed no transcripts to be significantly differentially expressed by 4 weeks of chronic IL-5 stimulation (10 ng/mL) (Figure 2A), and 24 h of acute IL-5 stimulation (10 ng/mL)induced only a decrease in the transcript for *PIAS4*, protein inhibitor of activated STAT4 (Figure 2B). Further, there were no transcriptomic differences in the trajectory of EpC differentiation (Figures S2A and S3A) among all groups. Similarly, neither acute nor chronic IL-5 stimulation showed any significant effect on TEER resistance measurements over the 4 weeks in culture (Figure 2C). To validate our functional assays for ALI cultures, we included IL-4 as a positive control, as it has been shown to induce alterations in intercellular junction proteins, reflecting increased epithelial permeability.^7^ IL-5 stimulation also had no effect on the self-tissue repair process, measured as rate of wound closure (Figure 2D). Our study aimed to parallel the methods used by the authors in the 2020, Barretto et al. study of bronchial EpCs in order to determine if, using these same in vitro conditions, sinonasal EpCs would also express functional IL-5Rα and CSF2RB and we found that they do not. However, there may well be inflammatory scenarios in vivo that could induce the expression of CSF2RB in these cells, and thus provide a situation in which the two IL-5 receptors were co-expressed on sinonasal EpCs, allowing for IL-5, which is often present at high levels in the milieu of the inflamed upper respiratory tract, to cause relevant immunologic consequence on these cells. Nonetheless, our current results, which also showed no discernible *CSF2RB* transcript expression on the in vivo ciliated EpCs, and only very minimal *CSF2RB* expression on the basal, apical, and glandular EpCs within the highly inflammatory milieu of CRS patients, suggest that sinonasal EpCs may not be a meaningful target of IL-5.

The discrepancy between our findings of lack of IL-5Rα and CSF2RB co-expression in sinonasal tissue EpCs compared to prior studies demonstrating functional co-expression of these receptors in bronchial EpCs warrants further investigation and raises questions about the roles of IL-5 and its receptors in the upper vs lower respiratory tracts. Anecdotally, clinicians treating patients in our aspirin-exacerbated respiratory disease (AERD) center at the Brigham and Women’s Hospital had suggested that their AERD patients with comorbid asthma and CRSwNP who were treated with the anti-IL-5 biologic mepolizumab would often report a perceived therapeutic benefit for their asthma symptoms and control, but a perceived lack of therapeutic benefit of mepolizumab on their sinus and nasal polyp-related symptoms. To better investigate this, we surveyed 89 AERD patients with asthma and comorbid CRSwNP who were treated with mepolizumab for at least 3 months, with the questions “Did mepolizumab improve your asthma symptoms?”, and “Did mepolizumab improve your sinus/nasal polyp symptoms?”, using a visual analogue scale of perceived improvement (from 0 = “not at all” to 100 = “significantly”). There was a significant difference in perceived efficacy of the anti-IL-5 treatment, with much more benefit reported for asthma symptoms and less for sinonasal symptoms (Figure 2E). Although this suggests potential differences in responsiveness to anti-IL-5 treatment between the upper and lower respiratory tracts, further prospective studies would be helpful as these data are based on purely patient-reported subjective criteria in patients with complex and heterogeneous diseases.

In conclusion, this study demonstrated that due to the lack of CSF2RB co-expression, IL-5 failed to induce significant changes in transcript expression, barrier integrity, or wound repair in cultured sinonasal EpCs. Though there may be relevant consequences of the binding of IL-5 to its IL-5Rα in upper respiratory EpCs, none are currently apparent. In fact, perhaps one explanation for why mepolizumab and benralizumab seem to be more efficacious for the treatment of asthma than they are for CRSwNP, despite intense respiratory tissue eosinophilia in both of those diseases, is that there is an additional role for IL-5 signaling in the EpCs of the bronchial airways that is absent in the EpCs of the upper airways.

## Supplementary Material

Supplemental Figures and Methods

Additional supporting information can be found online in the Supporting Information section at the end of this article.

## Data Availability

The data that support the findings of this study are available from the corresponding author upon reasonable request.
