# Supplementary material for: Human nasal epithelial cells express IL-5Rα but not the co-receptor CSF2RB and do not signal to IL-5
Source: Allergy. Author manuscript; Available in PMC 2025 Oct 1. (PMC12487116; doi:10.1111/all.16218)
Supplement: Supplemental Figures and Methods [file NIHMS2111098-supplement-Supplemental_Figures_and_Methods.pdf]

Supplementary Material:

Materials & Methods.

Patient characteristics and sample collection.

Subjects between the ages of 23 and 73 years with nasal polyps (CRSwNP) and healthy controls with no sinus disease were recruited from Brigham and Women's Hospital. All patients signed informed consent and these studies were approved by the MassGeneralBrigham Investigational Review Board.

Nasal polyp samples were collected during elective endoscopic sinus surgery, whereas healthy control samples were either collected during a transsphenoidal approach to pituitary surgery or from nasal brushings. Surgical samples were immediately put in R10 media before being transported back to undergo cell digestion. The tissue was cut up until there were no visibly large chunks and subsequently incubated and centrifuged twice for 15 minutes at 37°C in a digestion buffer of R10, collagenase, and DNase. Between incubation steps, the mixture was passed through a 20 mL syringe with a 16 gauge needle to produce finer tissue pieces. This step was repeated after a second incubation with an 18 gauge needle. The cell suspension was passed over a 70µM filter and centrifuged at 350g for 10 minutes. If any visible red blood cells were present, the pellet was reconstituted in red blood cell lysis buffer and EDTA and further centrifuged at 500g for 5 minutes.

Nasal brushings were collected using a CytoSoft Cytology brush (Fisher Scientific, Waltham MA). After scraping the nasal cavity 5-6 times, brushes were vortexed and placed into a 0.2 µM Corning® Costart Spin Filter that was then centrifuged for 20 minutes at 16,000g.

Cell culture.

Cell pellets formed from both tissue digestion and nasal brushings were immediately cultured in flasks coated in collagen with PneumaCult Ex Basal Media (StemCell, Vancouver, BC, Canada) supplemented with hydrocortisone (StemCell), penicillin-streptomycin (Sigma Aldrich, St. Louis, MO), and gentamicin and amphotericin B (Lifeline, Oceanside, CA). Primary nasal epithelial cells (EpCs) adhered to the bottom of the flask and were cultured through two passages that typically lasted for two weeks. After reaching confluency, cells were transferred to 12 Costar 6.5 mm Transwell Pore Polyester Membrane Inserts (StemCell) in a 24-well Air-Liquid Interface (ALI) plate at a density of 150K to 200K cells per 200µL of Ex Basal Media. Cells were seeded at the apical chamber of the transwells, while 600µL of Ex Basal Media was added to the basal chamber. After 2-3 days, cells were airlifted by removing media from the apical chamber. Ex Basal Media in the basal chambers were replaced with PneumaCult ALI Complete Media (StemCell) supplemented with ALI Maintenance Supplement (StemCell), Hydrocortisone (StemCell), and Heparin (StemCell). Media in the basal chamber was replaced every other day, and the cell culture was maintained for four weeks. During this period, two wells were chronically stimulated with IL-5 (R&D System, Minneapolis, MN), while another pair received stimulation with IL-4 (PeproTech, Cranbury, NJ) at a concentration of 10 ng/mL. Differentiation into a mucociliary epithelium became apparent around three weeks as signs of mucus appeared on the surface layer of the cells. After four weeks, cells were collected for RNA isolation (qPCR and RNAseq) and western blot.

qPCR.

RNA was extracted from epithelial cells collected before (T0) and after growing in the ALI culture for four weeks (T4) using the RNeasy Mini Kit (Qiagen, Germantown, MD). Complementary DNA was synthesized in a 10μL reaction with 200 ng of RNA and PrimeScript RT Master Mix (Takara. San Jose, CA) that was further diluted to 5 ng/μL. Quantitative polymerase chain reaction was performed using the Fast SYBR Green (Thermo Fisher) and specific primers including the soluble and membrane IL-5Rα isoforms, the common IL-5Rα, and CSF2RB (primers all from Qiagen), all of which were normalized to 18S (Qiagen).

Western Blot.

Protein from epithelial cells collected before (T0) and after four weeks of ALI culture (T4) were extracted and diluted with RIPA lysis buffer (Sigma-Aldrich), Protease Inhibitor cocktail (Sigma-Aldrich), and SDS Sample Buffer. Protein samples were then subjected to SDS gel electrophoresis and transferred to a blotting membrane (Millipore Sigma). Membranes were blocked with 5% milk and incubated overnight with anti-IL-5Rα (1:2,000) or CSF2RB (1:1,000) primary antibody. Membranes were washed with TBST and incubated for one hour with their respective anti-goat or anti-rabbit secondary antibodies.

Transepithelial resistance and wound repair.

Transepithelial electrical resistance (TEER) was measured with EVOM2 Epithelial Volt/Ohm Meter (World Precision Instruments, Sarasota, FL) for a duration of four weeks. TEER values (ohms) were calculated by subtracting from a blank value and multiplying by the area of the membrane (cm^2).

Following a previously published protocol (1), differentiated ALI cultures at week 4 were stimulated with or without IL-4 and IL-5 in hydrocortisone-deprived media three days prior to wounding. Using a P10 sterile pipette tip, cells were scraped in linear motion, leading to an injury. The apical surface of the ALI cultures was washed 3x with 200 μL PBS to remove cellular debris. Wound progression was monitored via microscopy at 4x magnification, capturing images every 6 hours. Image processing program ImageJ was used to track the wound area over time. Wound area was calculated each hour using ImageJ by converting pixels to area. The wound closure rate was quantified by computing the ratio of the closed wound area to the elapsed time in hours.

Bulk RNA sequencing.

RNA Extraction and Library Preparation. Total RNA was extracted from EpCs using the Qiagen RNAeasy kit. Subsequently, RNA sequencing was performed using the SmartSeq2 technology at the Genomics Platform, Broad Institute, Boston, MA. Briefly, RNA purification involved a 2.2X RNA-SPRI bead cleanup step. Complementary DNA (cDNA) synthesis from RNA was carried out using Maxima RNaseH-minus RT (ThermoFisher Scientific). The resulting cDNA underwent PCR pre-amplification with Kapa HiFi HotStart ReadyMix (KAPA Biosystems) and ISPCR Primer (IDT Technologies) to amplify cDNA molecules. Construction of cDNA libraries was achieved using the Nextera XT DNA Library Preparation kit (Illumina). The quality of both cDNA and libraries was assessed. Pooled libraries were then sequenced with a 2x38 read structure following Illumina's manufacturer protocols on the NextSeq platform.

Data Processing and Alignment. Unaligned and non-demultiplexed FASTQ files were processed, and reads were aligned to the Homo sapiens hg38 reference genome using the STAR aligner (version 2.6.1d)(2). Additional transcript-level information was obtained from Ensembl release 94. Quality control of sequencing data was conducted using FastQC (version 0.11.8) and Samtools (version 1.9), with results summarized using MultiQC (version 1.9). BAM files generated from the STAR aligner were further summarized using the featureCounts function from the Subread package.

Normalization and Differential Expression Analysis. Raw counts were subjected to normalization using DESeq2 (3) to account for differences in sequencing depth and variability due to low counts. Prior to normalization, transcripts with low counts <100 in all 90 samples were filtered out. Following normalization, differential expression analysis was performed in a pairwise manner with independent filtering to identify significantly upregulated genes in CRSwNP EpCs compared to healthy control samples, as well as in IL-5 stimulated samples compared to unstimulated samples. This analysis included a Wald test for pair-wise comparisons and a likelihood ratio test for multiple comparisons, often used in time-series data analysis. DESeq2 output provided nominal p-values, P-adjusted values (adjusted for multiple testing using the Benjamini–Hochberg procedure), and fold change values. Visualization of differential expression results was done using the "EnhancedVolcano" package, which included volcano plots.

Cell-Type Signature Analysis. To develop cell-type signatures, marker lists from published single-cell RNA sequencing (scSeq) studies(4) were used as a basis. To generate scores reflecting these signatures, RNA-sequencing count data were first normalized using DESeq2's variance-stabilizing transformation. The gene signature scores were then calculated using the UCell package, based on the Mann-Whitney U statistic.

Clinical response to anti-IL-5 treatment. Study subjects were adults with physician-diagnosed AERD who consented to participate in our AERD registry between 2013-2023, and who had comorbid asthma and CRSwNP. The MassGeneralBrigham Institutional Review Board approved the study. At registry enrollment, demographic information and AERD-related medical history are collected with surveys completed and stored in Research Electronic Data Capture (REDCap; Nashville, Tenn). Upon enrollment into the registry, patients are asked whether they have ever been treated with mepolizumab for at least 3 months, and if so, are further asked the questions “Did mepolizumab improve your asthma symptoms?”, and “Did mepolizumab improve your sinus/nasal polyp symptoms?” and are provided a visual analogue sliding scale of perceived improvement (from 0= “not at all” to 100= “significantly”). 89 patients with AERD who had comorbid CRSwNP and asthma had answered these survey questions at the time of this study.

Table S1. Patient characteristics. The following table lists the demographic data for the participants from whom samples were collected and analyzed for this study.

Characteristic	Healthy control (n = 16)	CRSwNP (n= 18)
Age (y), Mean ± SD	35.4 ± 11.9	43.2 ± 15.2
Sex (Female) n, (%)	12 (75%)	8 (44%)
Race (White) n, (%)	11 (68 %)	14 (77%)

Figure S1. Bulk RNA sequencing of epithelial cells cultured from excised sinus tissue from healthy and nasal polyps patients. Combined data from both patient groups is shown for expression of **(A)** *IL5RA*, **(B)** *FOXJ1*, and **(C)** *CSF2RB* over 4 weeks in culture with or without IL-5, and following acute stimulation with IL-5 x24 hours. All data points are shown, with boxes showing median and interquartile range.

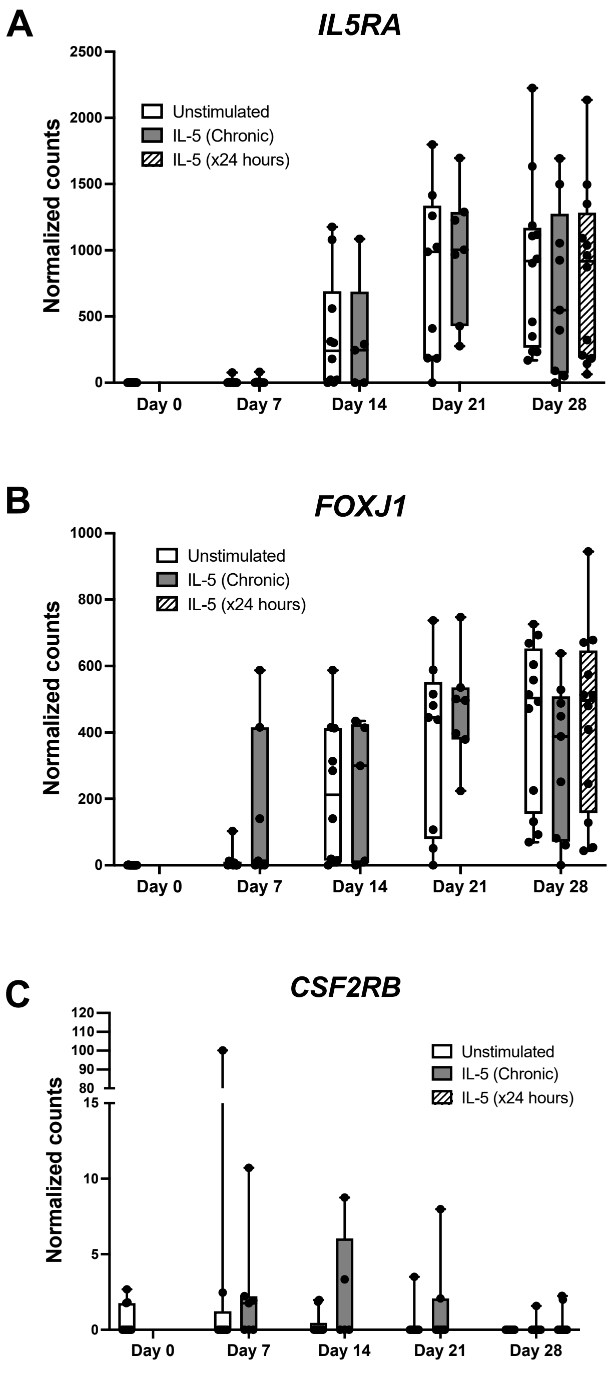

Figure S2. Bulk RNA sequencing of sinonasal epithelial cells cultured from excised sinus tissue from healthy and CRSwNP patients. (A) Principal component analysis (PC) plot of the variance between timepoint and disease. **(B)** Volcano plot of differentially expressed genes between CRSwNP vs healthy control from submerged cultures (left) and after 4 weeks of air-liquid interface differentiation (right).

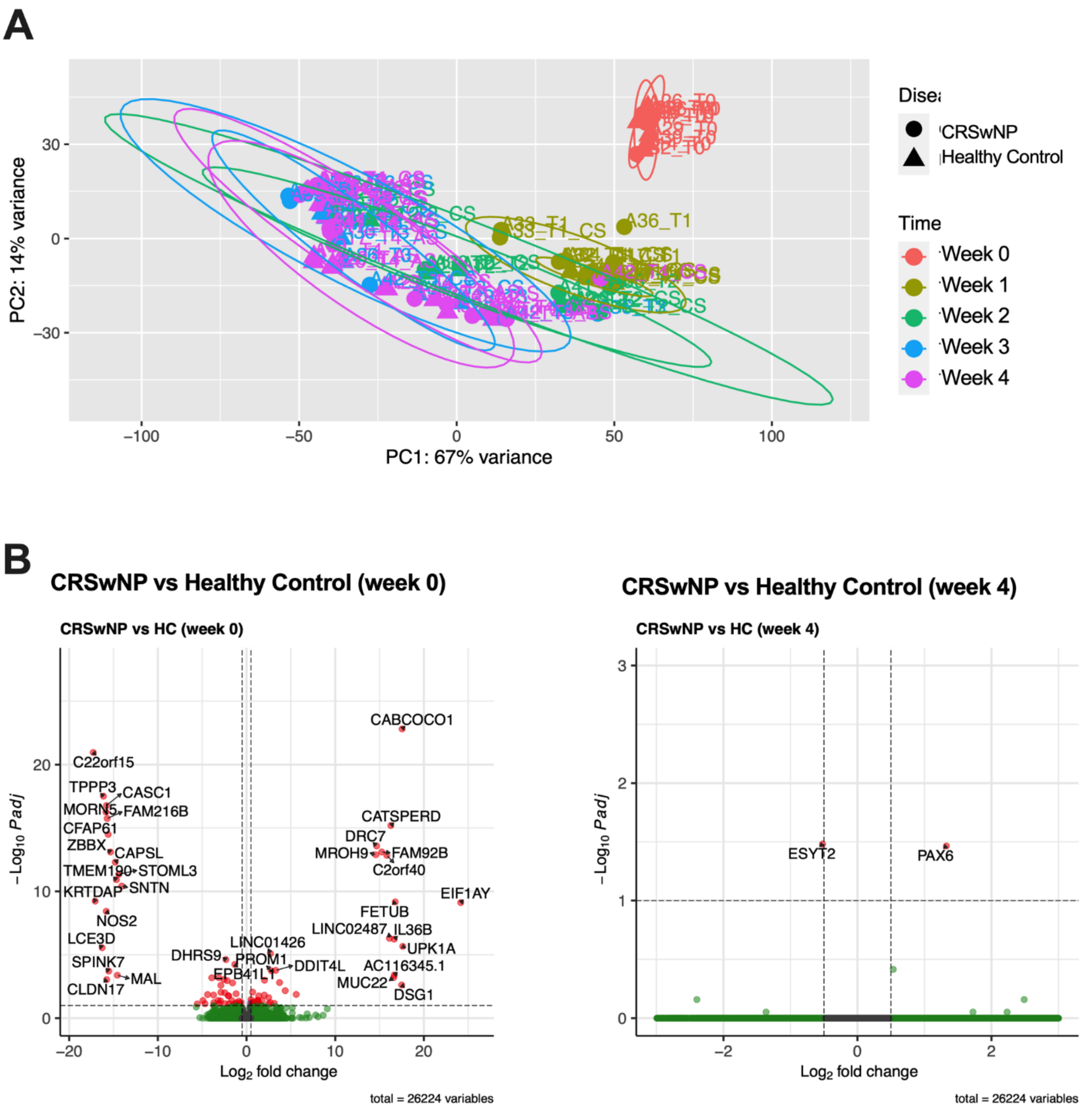

Figure S3. Cell-type gene signatures estimated from transcriptome profile: Cell-type gene signatures generated from single-cell RNA sequencing were applied to bulk RNA-sequenced epithelial cells from healthy or CRSwNP patients over time, both with and without IL-5 stimulation. Data shown are mean ± standard error.

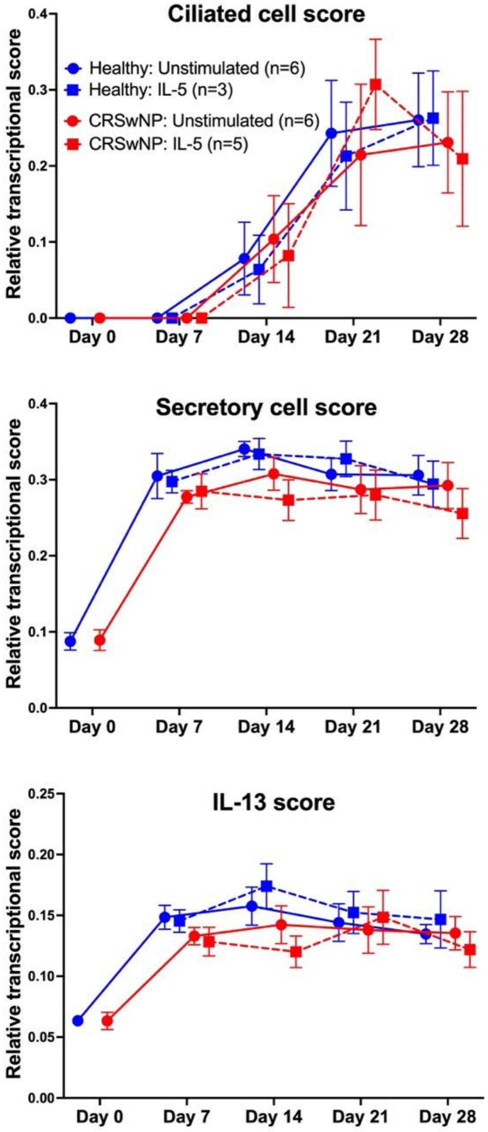

References.

1. Schagen J, Sly PD, Fantino E. Characterizing well-differentiated culture of primary human nasal epithelial cells for use in wound healing assays. Lab Invest. 2018;98(11):1478-86.
2. Dobin A, Davis CA, Schlesinger F, Drenkow J, Zaleski C, Jha S, et al. STAR: ultrafast universal RNA-seq aligner. Bioinformatics. 2013;29(1):15-21.
3. Love MI, Huber W, Anders S. Moderated estimation of fold change and dispersion for RNA-seq data with DESeq2. Genome Biol. 2014;15(12):550.
4. Kotas ME, Patel NN, Cope EK, Gurrola JG, 2nd, Goldberg AN, Pletcher SD, et al. IL-13-associated epithelial remodeling correlates with clinical severity in nasal polyposis. J Allergy Clin Immunol. 2023;151(5):1277-85.
